# Estimation of German KIR Allele Group Haplotype Frequencies

**DOI:** 10.3389/fimmu.2020.00429

**Published:** 2020-03-12

**Authors:** Ute V. Solloch, Daniel Schefzyk, Gesine Schäfer, Carolin Massalski, Maja Kohler, Jens Pruschke, Annett Heidl, Johannes Schetelig, Alexander H. Schmidt, Vinzenz Lange, Jürgen Sauter

**Affiliations:** ^1^DKMS, Tübingen, Germany; ^2^DKMS Life Science Lab, Dresden, Germany; ^3^University Hospital Carl Gustav Carus, Dresden, Germany

**Keywords:** KIR, HSCT, haplotype frequency, donor selection, immunogenetics

## Abstract

The impact of the highly polymorphic Killer-cell immunoglobulin-like receptor (*KIR*) gene cluster on the outcome of hematopoietic stem cell transplantation (HCST) is subject of current research. To further understand the involvement of this gene family into Natural Killer (NK) cell-mediated graft-versus-leukemia reactions, knowledge of haplotype structures, and allelic linkage is of importance. In this analysis, we estimate population-specific *KIR* haplotype frequencies at allele group resolution in a cohort of *n* = 458 German families. We addressed the polymorphism of the *KIR* gene complex and phasing ambiguities by a combined approach. Haplotype inference within first-degree family relations allowed us to limit the number of possible diplotypes. Structural restriction to a pattern set of 92 previously described *KIR* copy number haplotypes further reduced ambiguities. *KIR* haplotype frequency estimation was finally accomplished by means of an expectation-maximization algorithm. Applying a resolution threshold of ½ *n*, we were able to identify a set of 551 *KIR* allele group haplotypes, representing 21 *KIR* copy number haplotypes. The haplotype frequencies allow studying linkage disequilibrium in two-locus as well as in multi-locus analyses. Our study reveals associations between *KIR* haplotype structures and allele group frequencies, thereby broadening our understanding of the *KIR* gene complex.

## Introduction

The Killer-cell immunoglobulin-like receptor (*KIR*) gene cluster is located on the long arm of human chromosome 19 (19q13.4). The family includes the 15 expressed genes *KIR2DL1-4, KIR2DL5A, KIR2DL5B, KIR3DL1-3, KIR2DS1-5*, and *KIR3DS1* and the two pseudogenes *KIR2DP1* and *KIR3DP1* (1). *KIR* genes are characterized by a high level of sequence homology among different gene loci on one hand, and multi-layer complexity at the gene and haplotype level on the other hand (2, 3). The *KIR* gene copy number (CN) of a locus varies from 0 to 3 per haplotype (4). Each of the *KIR* loci displays high allelic polymorphism. One thousand one hundred ten allele variants and 543 KIR proteins have been named so far [Immuno Polymorphism Database IPD-*KIR* v2.9.0, December 2019 (5)]. In addition, diversity of the KIR gene region was found to be shaped in a population-specific manner through evolutionary mechanisms (6). Frequent occurrence of hybrid *KIR* genes (7–14), as well as events of alternative splicing of *KIR* genes, resulting in potentially functional receptor isoforms (15), have been described.

The high genetic complexity may be rooted in the diverse functions of *KIR* gene products in the human innate immune system. KIR proteins are transmembrane receptors primarily expressed by Natural Killer (NK) cells. Most KIR are located in the NK cell plasma or endosomal membrane and are known to be involved in the regulation of the immune response to viral infections and cancer or in the governance of histocompatibility during pregnancy (16–18). Bound to their corresponding ligands, which include classical and non-classical human leukocyte antigen (HLA) class I molecules, they convey activating or inhibitory signals to the NK cell (18, 19). *KIR3DL1-3, KIR2DL1-3*, and *KIR2DL5A/B* are the KIR receptors with inhibitory potential, the activating receptors are KIR3DS1 and KIR2DS1-5. The integrated signals of KIR and further NK cell receptors regulate NK cell activity between the two extremes of tolerance and killing (16). *KIR2DL4* plays a special role in the *KIR* gene family. This receptor appears to participate in the NK-mediated control of the maternal/fetal interface during pregnancy and is probably not involved in the cancer or infection surveillance (20, 21). However, lack of the *KIR2DL4* in maternal NK cells is still compatible with successful pregnancy (22).

In present-day unrelated hematopoietic stem cell transplantation (HSCT) practice, the alleles of particular *HLA* genes are matched between patient and stem cell donor to avoid undesired immune reactions of donor T-cells against the host organism (23–25). However, as graft-versus-host disease (GvHD) and disease relapse remain serious complications for many patients, there is an ongoing quest to identify further immunogenic factors with the potential to improve the outcome of HSCT. Since NK cells are the first lymphocytes to reconstitute in patients after HSCT (26), efforts have been made to exploit their potential to elicit a rapid and targeted immune response against remaining leukemic cells (27–32). Recent research indicates an impact of donor *KIR* genotype on the outcome of HSCT, but results remain controversial (33–37).

The knowledge of population-specific allelic haplotype frequencies for *HLA* genes of the major histocompatibility complex (MHC) offered advantages in the field of unrelated donor HSCT. For instance, all major donor search algorithms utilize *HLA* haplotype frequencies to estimate the probabilities for listed donors with ambiguous or incomplete *HLA* typing data to be a match for a specific patient (38, 39). Matching probability analyses using population-specific *HLA* haplotype frequencies allow targeted planning of donor center recruitment strategies (40). Beyond that, a positive impact on outcome of HSCT is proposed for the additional matching of non-*HLA* genes located in the MHC region via haplotype matching between patient and donor, but study results are still inconclusive (41–43).

Efficient use of *KIR* genes in donor selection for successful HSCT accordingly would benefit from knowledge of population-specific *KIR* haplotype frequencies. However, compared to *HLA, KIR* haplotype inference from separately typed loci is much more difficult because of the high number of possible diplotypes one unphased *KIR* genotype is compatible with. For example, two alleles genotyped at one *KIR* locus may be located either both on the same chromosome or each alone on one of the two chromosomes. These ambiguities and the high number of *KIR* loci lead to a large amount of possible allelic diplotypes that requires unrealistically large memory for the implementation of a conventional expectation-maximization (EM) algorithm.

A way to cope with the high number of possible allelic diplotypes per individual and to still reproduce the polymorphic nature of *KIR* haplotypes is to limit the underlying haplotype structure to pre-established patterns (44, 45). Previous research analyzed the haplotype structure of the *KIR* gene complex at various levels of resolution. Presence or absence of certain *KIR* loci in individuals was found not to occur at random, but to follow structural patterns. At the level of gene content (presence/absence, P/A) polymorphism, two major groups of *KIR* haplotypes (A and B) are described (2, 19). A and B haplotypes are characterized and distinguished by the presence of specific sets of *KIR* genes alongside the four “framework” genes (*KIR3DL3, KIR3DP1, KIR2DL4*, and *KIR3DL2*), flanking the centromeric and telomeric regions of the vast majority of all *KIR* haplotypes. *KIR2DS4* is the only activating receptor encoded by A haplotypes, whereas B haplotypes carry up to 5 activating KIR. More detailed analyses of *KIR* haplotypes and their frequencies in different cohorts were undertaken at gene content level (45–48), copy number level (4, 14, 45, 49, 50) and at (partial) allelic resolution level (44, 48, 49, 51, 52). All studies confirmed the basic concept of A/B haplotypes, but also documented numerous deviations from these structural patterns, caused, e.g., by recombination, gene fusion, deletion, or insertion events.

In our study, we extended the method to limit haplotype structure to pre-established patterns in order to make it applicable to our data obtained by high-throughput *KIR* genotyping in the context of unrelated HSCT donor registration (53, 54). We applied a three-step approach to estimate population-specific *KIR* haplotype frequencies at allele group resolution. We analyzed the *KIR* genotypes in a cohort of *n* = 458 families in order to reduce phasing and typing ambiguities and thus the number of possible diplotypes for the *n* = 916 parents. Structural complexity was further confined by restricting possible haplotypes to a set of 92 previously described *KIR* copy number haplotypes. Haplotype frequencies were then derived using an implementation of the EM algorithm that dealt with remaining ambiguities.

## Materials and Methods

### *KIR* and *HLA* Genotyping

Between October 2016 and April 2019, 2.6 million donors recruited by DKMS were *KIR* genotyped at allelic resolution by DKMS Life Science Lab in Dresden, Germany, using next generation sequencing methods (53, 55, 56). Genotyping comprises the *HLA* loci *HLA-A, -B, -C, -DRB1, -DQB1*, and -*DPB1*, the *KIR* loci *KIR2DL1-5, KIR3DL1-3, KIR2DS1-5, KIR3DS1, KIR2DP1*, and *KIR3DP1*, as well as *ABO* (57), *RhD, CCR5* (58), and *MIC-A/B* (59). The KIR genotyping approach delivers both allele group information and copy numbers for every KIR gene (53). DNA was extracted from blood samples or buccal swabs with the informed consent of the donors.

For the analysis of *KIR* haplotypes, *KIR* genotyping results were shortened to the first three digits of the allele name, thereby merging alleles with synonymous substitutions within the coding region and non-coding mutations. Allelic ambiguities due to variations outside the typed exons (exons 3, 4, 5, 7, 8, and 9) were grouped and denoted by a trailing “c” (Supplementary Information S1). In the following, 3-digit allelic level and “c”-groups are referred to as “allele group” resolution. Since a clear distinction of genes of the loci *KIR2DL5A* and *KIR2DL5B* was not possible without sequence information on exon 1 and promoter region, we treated *KIR2DL5A* and *KIR2DL5B* as one locus. We considered 16 *KIR* loci in total.

### Family Selection

Information on family relations is not recorded during DKMS donor recruitment. Data retrieval from the DKMS Germany donor file, demanding consistency of addresses and surnames and a minimal age difference of >20 years between parents and offspring, yielded a pseudonymized set of potential families of self-assessed German origin. The genetic relationship between potential family members was verified on the basis of the respective *HLA-A, -B, -C, -DRB1*, and *-DQB1* typing data. A cohort of 458 *HLA*-confirmed families with two parents and at least one child were included into our study. Four hundred two of the families had one child, 55 had two, and one family had three children registered with the donor center (*n*_*total*_ = 1,431).

### *KIR* Data Refinement via Family Information

*KIR* genes of all members of our family cohort were resolved to allele group level and copy number. Knowledge of family relations was used to review *KIR* typing when comparison of data between parents and children revealed ambiguities or inconsistencies. For instance, in 1.6% of the 14,656 typed parental *KIR* loci (916 samples × 16 loci), the allele group typing results of a child apparently did not match the parental genotypes. All but 13 cases, concerning in total 10 sets of parents, could be solved by re-inspection of the sequencing data and application of the family information. The matching status of new and so far unnamed alleles between the family members (464 cases) was verified by comparison of sequencing data and was accordingly considered during haplotype inference. Families with more than one child (*n* = 56) permitted investigation of potential recombination events by intersecting parental haplotypes deduced via *KIR* genotyping data of different children.

### Copy Number Haplotype Pattern Set (CNPS)

In order to reduce haplotype complexity originating from phasing ambiguities between *KIR* loci, only *KIR* genotypes that could be split into two haplotypes that met the structural pattern of 92 previously described *KIR* copy number haplotypes were permitted. The set of 92 *KIR* copy number haplotypes used as reference pattern is hereinafter referred to as CNPS. The CNPS included 12 copy number haplotypes described by Pyo et al. (48), 52 by Jiang et al. (4), 27 by Pyo et al. (50), and one by Roe et al. (14) (Supplementary Information S2). Because nomenclature of *KIR* haplotypes in the different publications in not consistent, we assigned a code name (column “HT code” in Supplementary Information S2) to every copy number haplotype in the CNPS which will be used below.

*KIR* loci *KIR2DL5, KIR2DS3*, and *KIR2DS5* have experienced a duplication event in the past and can be found in both the centromeric and the telomeric section of particular haplotypes of the *KIR* gene complex (60, 61). We could not distinguish between the respective centromeric and telomeric variants of the three loci in our analysis. For copy number haplotypes from publications where *KIR2DS3* and *KIR2DS5* were treated as one single locus and where thus the assignment to one of the loci was ambiguous, we split the haplotype into all possible copy number forms. For example, a haplotype which specifies 2 copies of locus *KIR2DS3S5* was split into three allowed copy number haplotypes: one haplotype with one copy of *KIR2DS3* and *KIR2DS5*, each, and two haplotypes with two copies of one of the two genes and none of the other. Copy number haplotypes marked as containing hybrid or fusion gene loci by Pyo et al. (50) and Roe et al. (14) were disregarded.

### Workflow

**Figure 1:**
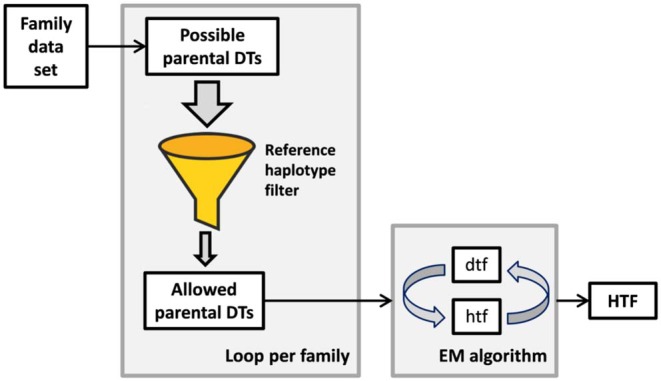
Simplified workflow of our haplotype frequency estimation approach. The number of 16-locus *KIR* diplotypes per individual is reduced by inference from a family context and subsequent filtering via a set of reference copy number haplotypes. Only diplotypes that can be explained by a pair of reference haplotypes are allowed. Remaining ambiguities are resolved in a conventional expectation-maximization (EM) algorithm. DT, diplotype; dtf, preliminary diplotype frequencies; htf, preliminary haplotype frequencies; HTF, final haplotype frequencies.

All software required for *KIR* haplotype frequency (HF) estimation in our approach was written in Perl 5. A schematic overview of the algorithm is shown in Figure 1. Families were analyzed in mother-father-child sets, i.e., a family with two children was analyzed in two separate family sets. Only diplotypes of the 916 parents were allowed to pass into the subsequent *KIR* HF estimation. Parents whose haplotypes had already counted for frequency estimation via a first family set were flagged to avoid duplication. For each of the mother-father-child constellations of our data set, all possible allele group diplotypes of mother and father were deduced. This was done by locus-wise diplotype inference and subsequent iteration of all possible allele constellations of all loci. The extensive diplotype list was then filtered with the CNPS to exclude all diplotypes that could not be explained by a pair of the allowed copy number haplotypes. In order to limit artifacts from lower-resolution typing data, we restricted the number of possible diplotypes per individual to a maximum of *n* = 1,000,000. Parents with more possible diplotypes after application of the CNPS were excluded from the subsequent HF estimation, unless they passed this requirement in a family constellation with another child. The most likely set of *KIR* HFs was finally derived from the parental diplotypes by means of an expectation-maximization (EM) algorithm (62). Haplotypes were initialized with equal frequencies prior to the start of the EM algorithm. The stop criterion, which defines the allowed maximal HF change between consecutive estimations, was set to 5^*^10^−5^. HFs were cut after a minimal frequency of *f* = 1/2*n*, with *n* being the number of individuals in the final haplotype estimation, corresponding to the occurrence of at least one haplotype in the sample. The resulting allele group haplotypes were attributed to the corresponding CNPS haplotypes in order to analyze the allelic diversity within the different copy number structures.

HFs were estimated both for the entire *KIR* gene region and separately for the centromeric and telomeric sections. In order to be able to assign the allele combinations of gene cluster *KIR2DL5*~*KIR2DS3*~*KIR2DS5* to a centromeric or telomeric location, we included these genes in both estimations of partial *KIR* HFs.

The approach to reduce the ambiguous nature of the *KIR* gene complex by means of the CNPS also permits the imputation of allelic haplotypes from large cohorts of individuals without a family context. In order to investigate the additional benefit of phase resolution via family affiliation, allele group diplotypes of all 916 parents of our cohort were determined in a separate workflow without the phasing knowledge of their families. Filtering of the diplotypes via the CNPS and estimation of the allele group *KIR* HF by the EM algorithm was carried out according to the family workflow described above.

### Linkage Disequilibrium

The linkage disequilibrium (LD) coefficient D' was calculated from the entire HF set (before cut below *f* = 1/2*n*) for any two-locus combination (63, 64). Significance was tested with Fisher's exact test. *P*-values were subjected to a Holm-Bonferroni correction for multiple testing. Multi-locus LD analysis was conducted using the normalized entropy difference ε between the observed HFs and those expected under the null hypothesis of linkage equilibrium (64, 65). The value of ε ranges between 0 and 1, with larger values indicating stronger LD. An absent locus (“NEG”) was treated as one allele variant.

## Results

### *KIR* Genotyping Resolution of the Family Cohort

91.8% of the 22,896 (1,431 donors × 16 loci) typed loci in our final *KIR* family data set were determined to allelic resolution, absence, or allele group level. Of this share, 74.7% of the identified copy number equivalents were resolved to allelic level, 21.5% to absence of the locus and 3.8% to broader allele groups, where remaining ambiguities impeded the differentiation between two or more alleles.

The remaining 8.2% of the 22,896 typed loci carried ambiguities either due to unknown phasing of the typed exons (e.g., KIR2DL1^*^003c + KIR2DL1^*^004|KIR2DL1^*^006 + KIR2DL1^*^010) or to uncertain copy number determination (e.g., KIR2DL3^*^001 + KIR2DL3^*^002| KIR2DL3^*^001 + KIR2DL3^*^001 + KIR2DL3^*^002), allowing for multiple valid allele (group) pairs.

### Parental Cohort

In the final analysis, *KIR* data of 790 of the 916 parents was used to estimate HFs of the entire *KIR* gene region. The 790 individuals originate from 403 of the 458 families. Both parents were included into the HF estimation for 387 families, 16 further families contributed one parent, each.

Reasons for exclusion from the final analysis set were: 97 parents (10.6%) were excluded because no combination of the allowed 92 copy number haplotypes could explain the deduced diplotypes. Twenty parents (2.2%) were excluded because of mismatching to all of their potential children in one *KIR* locus (nine families) or two *KIR* loci (one family). Seven further parents (0.8%) were excluded because the number of possible diplotypes after the filtering step exceeded the threshold (*n* = 1,000,000) of possible *KIR* diplotypes per individual. Finally, both parents (0.2%) of one family were omitted because the respective diplotype estimations on the basis of the *KIR* data of two children showed no intersection, but indicated a possible recombination event in the father's chromosomes (Supplementary Information S3). In this case, comparison of the father's different haplotype sets derived from two children revealed an exchange in alleles in locus *KIR3DL3*.

Consideration of only centromeric or telomeric genes for *KIR* haplotype estimation in the family context allowed the inclusion of more parents. HFs were calculated from KIR data of 838 (876) individuals for the centromeric (telomeric) loci. The number of individuals excluded because no combination of valid CNPS haplotypes could explain the diplotypes decreased to 6.6% (3.5%). Omission because of mismatching was reduced to 1.7% (0.7%), None of the parents were excluded due to high numbers of possible diplotypes.

When we deduced allele group diplotypes of the 916 parents for the entire *KIR* gene region without the phasing knowledge of the family relations, the parental cohort that could be included was considerably smaller. Applying the same filtering and configuration values as in the family approach, *KIR* data of only 438 (47.8%, compared to 790 or 86.2% in the family calculations) individuals passed into the HF estimation. On the one hand, only 6 individuals (0.7% vs. 10.6% in the family approach) were omitted because no valid combination of the reference copy number haplotypes could explain their diplotypes. On the other hand, however, the number of possible diplotypes exceeded the threshold of *n* = 1,000,000 in 473 parents (51.6 vs. 0.8% in the family approach), demonstrating the efficiency of ambiguity reduction via family information.

### *KIR* Haplotype Frequencies for the Entire Gene Region

**Figure 2:**
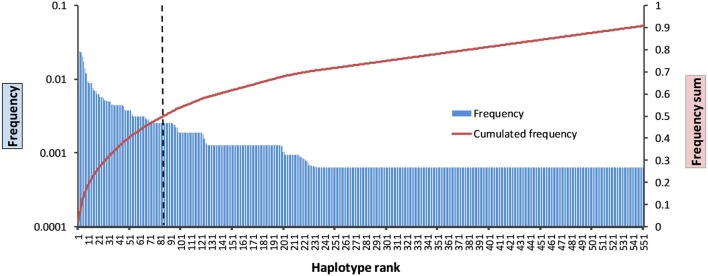
Frequency distribution of the 551 allele group *KIR* haplotypes above the resolution threshold of *f* = 1/2*n*. Blue bars: haplotype frequencies; red line: cumulated frequency; dashed black line: haplotype rank 84 with 50% cumulated haplotype frequency.

*KIR* HF estimation for the data of the 790 parents resulted in 551 different *KIR* allele group haplotypes with *f* ≥ 1/2*n*, corresponding to a frequency sum of 90.8% (Figure 2, Supplementary Information S4). The 20 most frequent allele group *KIR* haplotypes are listed in Table 1 and comprise a cumulated frequency of 26.0%.

**Table 1:** Top 20 allele group *KIR* haplotypes ranked by their respective frequencies.

**A/B nomenclature**	**HT code**	**3DL3**	**2DS2**	**2DL2**	**2DL3**	**2DP1**	**2DL1**	**3DP1**	**2DL4**	**3DL1**	**2DS4**	**3DS1**	**2DL5**	**2DS3**	**2DS5**	**2DS1**	**3DL2**	**Frequency**
cB02~tA01	P10_08	**003**	**001c**	**003**	NEG	NEG	NEG	**001c**	**001**	**002**	**001c**	NEG	NEG	NEG	NEG	NEG	**002c**	0.029046
cA01~tA01	P10_01	**001**	NEG	NEG	**002**	**003**	**001c**	**006**	**008**	**004**	**006**	NEG	NEG	NEG	NEG	NEG	**005**	0.023675
cA01~tA01	P10_01	**002**	NEG	NEG	**001**	**NEW**	**003c**	**001c**	**011**	**005**	**010**	NEG	NEG	NEG	NEG	NEG	**001c**	0.023363
cA01~tA01	P10_01	**001**	NEG	NEG	**001**	**002**	**003c**	**001c**	**001**	**015c**	**001c**	NEG	NEG	NEG	NEG	NEG	**002c**	0.022692
cA01~tA01	P10_01	**009**	NEG	NEG	**001**	**NEW**	**003c**	**001c**	**011**	**005**	**010**	NEG	NEG	NEG	NEG	NEG	**001c**	0.020308
cA01~tA01	P10_01	**008**	NEG	NEG	**001**	**005**	**003c**	**001c**	**008**	**001c**	**003**	NEG	NEG	NEG	NEG	NEG	**001c**	0.017089
cB01~tB01	P10_06	**003**	**001c**	**001**	NEG	**001**	**004**	**001c**	**005**	NEG	NEG	**013c**	**002c+002c**	**001c+002**	NEG	**002c**	**007**	0.014089
cA01~tA01	P10_01	**001**	NEG	NEG	**002**	**003**	**001c**	**NEW**	**001**	**002**	**001c**	NEG	NEG	NEG	NEG	NEG	**002c**	0.012025
cA01~tA01	P10_01	**009**	NEG	NEG	**001**	**002**	**003c**	**001c**	**011**	**005**	**010**	NEG	NEG	NEG	NEG	NEG	**010**	0.012025
cA01~tA01	P10_01	**013**	NEG	NEG	**002**	**003**	**001c**	**006**	**008**	**004**	**006**	NEG	NEG	NEG	NEG	NEG	**009**	0.009494
cA01~tA01	P10_01	**013**	NEG	NEG	**002**	**003**	**001c**	**NEW**	**006**	**007c**	**004**	NEG	NEG	NEG	NEG	NEG	**008**	0.008861
cA01~tA01	P10_01	**019**	NEG	NEG	**002**	**003**	**001c**	**006**	**008**	**004**	**006**	NEG	NEG	NEG	NEG	NEG	**005**	0.008861
cA01~tA01	P10_01	**001**	NEG	NEG	**002**	**003**	**001c**	**NEW**	**008**	**001c**	**003**	NEG	NEG	NEG	NEG	NEG	**001c**	0.008861
cA01~tA01	P10_01	**002**	NEG	NEG	**001**	**002**	**003c**	**001c**	**008**	**001c**	**003**	NEG	NEG	NEG	NEG	NEG	**001c**	0.008769
cA01~tB01	P10_03	**001**	NEG	NEG	**001**	**002**	**003c**	**001c**	**005**	NEG	NEG	**013c**	**001c**	NEG	**002**	**002c**	**007**	0.007493
cA01~tA01	P10_01	**001**	NEG	NEG	**002**	**003**	**001c**	**006**	**011**	**005**	**010**	NEG	NEG	NEG	NEG	NEG	**001c**	0.007104
cA01~tA01	P10_01	**002**	NEG	NEG	**001**	**005**	**003c**	**001c**	**008**	**001c**	**003**	NEG	NEG	NEG	NEG	NEG	**001c**	0.006962
cA01~tB01	P10_03	**009**	NEG	NEG	**001**	**002**	**003c**	**001c**	**005**	NEG	NEG	**013c**	**001c**	NEG	**002**	**002c**	**007**	0.006842
cB02~tA01	P10_08	**003**	**001c**	**003**	NEG	NEG	NEG	**001c**	**008**	**001c**	**003**	NEG	NEG	NEG	NEG	NEG	**001c**	0.006329
cA01~tA01	P10_01	**001**	NEG	NEG	**002**	**003**	**001c**	**005**	**001**	**002**	**001c**	NEG	NEG	NEG	NEG	NEG	**002c**	0.006329

*Framework genes are shaded in gray. Alleles of present loci are indicated in bold letters. A/B haplotype nomenclature according to Pyo et al. (48). HT code, code of the copy number haplotype pattern (see Supplementary Information S2); c, centromeric; t, telomeric; ~, point of separation between framework genes KIR3DP1 and KIR2DL4*.

Of the 92 permitted reference copy number haplotypes, only 21 are represented in the 551 estimated allele group haplotypes. Table 2 shows identity and frequencies of these CNPS haplotypes. Where the assignment was possible, the A/B haplotype nomenclature established by 48 is indicated. The most frequent of the CNPS haplotypes is P10_01 (cA01~tA01) with *f* = 59.6%.

**Table 2:** List of the 21 *KIR* copy number haplotypes represented in the 551 allele group *KIR* haplotypes above the resolution threshold and their frequencies.

**3DL3**	**2DS2**	**2DL2**	**2DL3**	**2DP1**	**2DL1**	**3DP1**	**2DL4**	**3DL1**	**2DS4**	**3DS1**	**2DL5**	**2DS3**	**2DS5**	**2DS1**	**3DL2**	**Frequency**	**HT code**	**A/B nomenclature**
1	0	0	1	1	1	1	1	1	1	0	0	0	0	0	1	0.59554	P10_01	cA01~tA01
1	1	1	0	0	0	1	1	1	1	0	0	0	0	0	1	0.09810	P10_08	cB02~tA01
1	0	0	1	1	1	1	1	0	0	1	1	0	1	1	1	0.07603	P10_03	cA01~tB01
1	1	1	0	1	1	1	1	1	1	0	1	1	0	0	1	0.05190	P10_04	cB01~tA01
1	1	1	0	1	1	1	1	0	0	1	2	2	0	1	1	0.02870	P10_06	cB01~tB01
1	1	1	0	1	1	1	1	0	0	1	2	1	1	1	1	0.01518	P10_07	cB01~tB01
1	1	1	0	0	0	1	1	0	0	1	1	0	1	1	1	0.01266	P10_10	cB02~tB01
1	0	0	1	1	1	1	1	0	0	1	1	1	0	1	1	0.01245	P10_02	cA01~tB01
1	1	1	0	0	0	1	1	0	0	1	1	1	0	1	1	0.00253	P10_09	cB02~tB01
1	1	1	0	1	1	2	2	1	1	1	1	1	0	0	1	0.00253	J12_13	cB^*^~t^#^
1	0	0	1	1	0	0	0	0	0	0	0	0	0	1	0	0.00253	J12_22	cA^*^~tB^*^
1	1	1	0	0	0	0	0	0	0	0	1	0	1	1	1	0.00190	J12_12	cB^*^~tB^*^
1	0	0	1	1	0	1	1	1	1	0	0	0	0	0	1	0.00127	J12_27	cA^*^~tA1
1	1	0	1	1	1	1	1	1	1	0	0	0	0	0	1	0.00127	J12_53	cB^*^~tA1
1	0	1	0	0	0	1	1	1	1	0	0	0	0	0	1	0.00127	J12_65	cA^*^~tA1
1	0	0	1	1	1	0	0	0	1	0	0	0	0	0	1	0.00063	P12_01	cA01~tA01-del5
1	0	0	1	1	1	2	2	0	0	2	1	0	1	1	1	0.00063	J12_28	cA^*^~tB^*^
1	1	1	0	0	1	1	1	1	1	0	0	0	0	0	1	0.00063	P12_04	cB01|tA01-del9
0	0	0	1	1	1	1	1	1	1	0	0	0	0	0	1	0.00063	J12_60	cA^*^~tA1
1	0	0	1	1	1	2	2	1	1	1	0	0	0	0	1	0.00063	J12_16	cA^*^~t^#^
1	1	1	0	0	0	2	2	1	1	1	0	0	0	0	1	0.00063	J12_30	cB02|tA01-ins4

Framework genes are shaded in gray. A/B haplotype nomenclature according to Pyo et al. (48, 50) was added where the assignment was possible. HT code, code of the copy number haplotype pattern; c, centromeric; t, telomeric; ~, point of separation between framework genes KIR3DP1 and KIR2DL4;

* , affiliation to a general A or B structure but deviation from the established nomenclature;

# *, distinction between A and B not possible*.

### Allelic Diversity

We attributed the allele group haplotypes to the respective copy number haplotypes in order to analyze the allelic diversity within the different copy number structures (Supplementary Information S5). However, due to the different frequencies of the copy number haplotypes and thus the different chances to detect allelic diversity, observations in our dataset can only be seen as indications of patterns. For the analysis, we considered only eight copy number haplotypes with a frequency of *f* ≥ 1% (Table 2). The number of allele group haplotypes per copy number haplotype varies between 8 and 318, with a clear positive correlation between copy number HF and the number of allele group haplotypes. All eight copy number haplotypes are combinations of only three centromeric (cA01, cB01, and cB02) and two telomeric (tA01 and tB01) motifs (in the nomenclature introduced by Pyo et al. (48).

**Figure 3:**
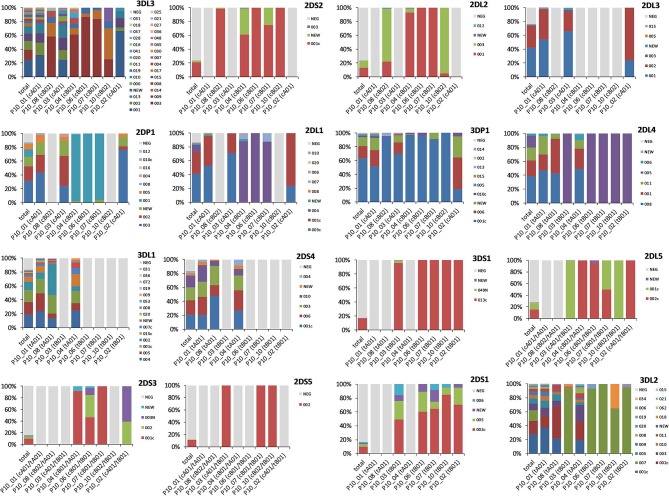
Allele group frequencies per *KIR* gene. Displayed are, for each of the 16 *KIR* genes, the allele frequencies for the estimated allele group KIR HF overall (total) and for the 8 CNPS haplotypes with a frequency of *f* ≥ 1%. The proportion of absence of the respective gene is indicated as “NEG” in light gray. The centromeric or telomeric haplotype structure of the respective CNPS haplotypes in A/B haplotype nomenclature according to Pyo et al. (48, 50) is indicated in brackets in the labeling of the diagram axes.

Figure 3 shows for each KIR gene the allele frequencies overall and for each copy number haplotype. Marked differences in overall allelic variability can be observed between the different genes with the highest diversities in the two outermost framework genes *KIR3DL3* (*n* = 29 named alleles) and *KIR3DL2* (*n* = 17), as well as in *KIR3DL1* (*n* = 14). Allelic variability and identity within one locus, however, depends clearly on the respective haplotype motif. Essentially, A haplotype motifs have a higher allelic diversity than B haplotype motifs in both, the centromeric and the telomeric *KIR* section. In addition, the different structural motifs also correlate with different alleles. For example, in locus *KIR3DL2*, tB01 haplotypes (P10_02, _03, _06, _07, and _10) are clearly dominated by allele ^*^007 with allele frequencies between 65.0 and 100%. tA01 haplotypes (P10_01, _04, and _08), in contrast, have a much higher allelic variability in *KIR3DL2* where allele ^*^007 only reaches a maximum frequency of 0.4%. Similar patterns can be seen in all framework genes and in genes *KIR2DP1* and *KIR2DL1*, which are present in both, A and B haplotypes.

The allocation of allele group haplotypes to the copy number haplotypes also reveals conserved allele combinations. For instance, the partial haplotype *KIR2DL2*~*KIR2DL3*~*KIR2DL1* that exhibited linkage disequilibrium also in the LD analysis (see below) displays an interesting pattern of allelic distribution. cB1 haplotypes (P10_04, _06, and _07) are almost exclusively populated with allelic haplotype block 001~NEG~004, while cA01 haplotypes (P10_01, _02, and _03) are dominated by allele group combinations NEG~001~003c and NEG~002~001c. Allele ^*^003 prevails in cB02 haplotypes (P10_08 and _10) with only *KIR2DL2* present. A second example is the gene cluster *KIR2DL5*~*KIR2DS3*~*KIR2DS5*. Our typing method does not allow the unambiguous assignment of this gene cluster to a centromeric or telomeric localization. However, the allele distribution of these genes in the different copy number haplotypes clearly reflects a conserved linkage of certain allele group combinations that reveal the position of the cluster in the *KIR* gene region. On the one hand, P10_04 (cB01~tA01), representing a centromeric localization of the cluster, is almost exclusively composed of KIR2DL5^*^002c~KIR2DS3^*^001c. A very small fraction of *KIR2DS3* alleles is “NEW”. On the other hand, tB01, the common haplotype structure of P10_02, P10_03, and P10_10, is known to include a telomeric *KIR2DL5*~*KIR2DS3*~*KIR2DS5* gene cluster. While the exclusive allelic composition in CN haplotypes P10_03 and P10_10 in our data is 2DL5^*^001c~2DS5^*^002, P10_02 carries either of the two combinations 2DL5^*^002c~2DS3^*^002 and 2DL5^*^002c~2DS3^*^003N. The conserved centromeric and telomeric allele combinations in our data set are listed in the Supplementary Information S6.

### Linkage Disequilibrium

LD coefficient D' was calculated for all two-locus haplotypes. Altogether, 368 pairs with significant deviation from equilibrium were identified (Supplementary Information S7), 129 of them with positive D'. In a large number of the significant cases of LD, one or both of the “alleles” is the absent locus. Thirty-two pairs of actual alleles in significant LD with *D'*≥ 0.9 and a HF of *f* ≥ 0.1 are listed in Table 3. Eleven of the pairs map to the centromeric section of the *KIR* gene complex, 18 to the telomeric section. The three pairs including loci *KIR2DL5* and *KIR2DS5* show close linkage to *KIR2DL4* and thus are probably located on the telomeric part of the *KIR* gene cluster.

**Table 3:** 2-locus linkage disequilibrium.

**Loci**	**Haplotype ab**	**f (ab) observed**	**f (a)**	**f (b)**	**D′**	**f (ab) expected**	***p***	***p*^**(HB)**^**
2DL1-3DP1	2DL1*001c-3DP1*006	0.1591	0.3080	0.1681	0.9225	0.0518	6.21E-13	2.33E-06
2DL2-2DP1	2DL2*001-2DP1*001	0.1038	0.1395	0.1095	0.9400	0.0153	4.78E-15	3.97E-06
2DL3-2DL1	2DL3*001-2DL1*003c	0.3794	0.4107	0.4004	0.9109	0.1645	1.43E-22	3.30E-06
2DL3-2DL1	2DL3*002-2DL1*001c	0.2916	0.3036	0.3080	0.9428	0.0935	1.05E-24	3.30E-06
2DL3-2DP1	2DL3*002-2DP1*003	0.2871	0.3036	0.3063	0.9214	0.0930	9.27E-24	3.35E-06
2DL3-3DP1	2DL3*002-3DP1*006	0.1661	0.3036	0.1681	0.9830	0.0510	3.36E-14	3.27E-06
2DL4-2DL5	2DL4*005-2DL5*001c	0.1078	0.1882	0.1134	0.9388	0.0213	1.30E-13	1.58E-06
2DL4-2DS1	2DL4*005-2DS1*002c	0.1056	0.1882	0.1127	0.9223	0.0212	4.05E-13	1.53E-06
2DL4-2DS4	2DL4*008-2DS4*006	0.1908	0.3646	0.1975	0.9466	0.0720	5.77E-13	1.54E-06
2DL4-2DS4	2DL4*001-2DS4*001c	0.1899	0.2106	0.1971	0.9532	0.0415	9.42E-22	1.54E-06
2DL4-2DS4	2DL4*011-2DS4*010	0.1595	0.1772	0.1620	0.9810	0.0287	1.19E-20	1.54E-06
2DL4-2DS5	2DL4*005-2DS5*002	0.1272	0.1882	0.1354	0.9252	0.0255	2.58E-15	1.55E-06
2DL4-3DL1	2DL4*008-3DL1*004	0.1737	0.3646	0.1791	0.9523	0.0653	6.61E-12	1.66E-06
2DL4-3DL1	2DL4*011-3DL1*005	0.1699	0.1772	0.1724	0.9822	0.0306	6.13E-22	1.66E-06
2DL4-3DL1	2DL4*008-3DL1*001c	0.1620	0.3646	0.1656	0.9656	0.0604	3.75E-11	1.66E-06
2DL4-3DL1	2DL4*001-3DL1*002	0.1039	0.2106	0.1104	0.9250	0.0233	6.27E-12	1.66E-06
2DL4-3DL2	2DL4*001-3DL2*002c	0.1705	0.2106	0.1794	0.9367	0.0378	2.10E-19	1.43E-06
2DL4-3DL2	2DL4*005-3DL2*007	0.1658	0.1882	0.1767	0.9241	0.0333	7.40E-20	1.43E-06
2DL4-3DS1	2DL4*005-3DS1*013c	0.1810	0.1882	0.1908	0.9524	0.0359	6.21E-22	1.64E-06
2DL5-2DS5	2DL5*001c-2DS5*002	0.1127	0.1134	0.1354	0.9925	0.0154	6.55E-17	1.21E-06
2DP1-2DL1	2DP1*002-2DL1*003c	0.1967	0.2070	0.4004	0.9165	0.0829	1.88E-11	2.80E-06
2DP1-3DP1	2DP1*003-3DP1*006	0.1572	0.3063	0.1681	0.9064	0.0515	1.00E-12	2.77E-06
2DS2-2DL2	2DS2*001c-2DL2*003	0.1023	0.2180	0.1057	0.9592	0.0230	1.83E-11	4.65E-06
2DS4-3DL2	2DS4*001c-3DL2*002c	0.1712	0.1971	0.1794	0.9429	0.0354	1.24E-20	1.10E-06
3DL1-2DS4	3DL1*004-2DS4*006	0.1766	0.1791	0.1975	0.9824	0.0354	1.15E-21	1.34E-06
3DL1-2DS4	3DL1*001c-2DS4*003	0.1634	0.1656	0.1810	0.9837	0.0300	3.67E-21	1.34E-06
3DL1-2DS4	3DL1*005-2DS4*010	0.1563	0.1724	0.1620	0.9574	0.0279	3.96E-20	1.34E-06
3DL1-2DS4	3DL1*002-2DS4*001c	0.1060	0.1104	0.1971	0.9501	0.0218	1.24E-12	1.34E-06
3DL1-3DL2	3DL1*002-3DL2*002c	0.1074	0.1104	0.1794	0.9662	0.0198	7.04E-14	1.26E-06
3DL3-2DL3	3DL3*002-2DL3*001	0.1203	0.1207	0.4107	0.9948	0.0496	1.54E-07	5.14E-05
3DL3-2DL3	3DL3*009-2DL3*001	0.1047	0.1082	0.4107	0.9455	0.0444	2.38E-06	5.17E-05
3DS1-2DS1	3DS1*013c-2DS1*002c	0.1039	0.1908	0.1127	0.9033	0.0215	7.11E-13	1.24E-06

*Shown are the 32 significant cases which fulfill the filtering criteria (D′ ≥ 0.9, f (ab) ≥ 0.1, both loci present). f (ab) = haplotype frequency; f (a) and f (b), frequency of allele (groups) a and b, respectively; D′, relative linkage disequilibrium; p, p-value (Fisher's exact test); p^(HB)^, p-value after Holm-Bonferroni correction*.

**Figure 4:**
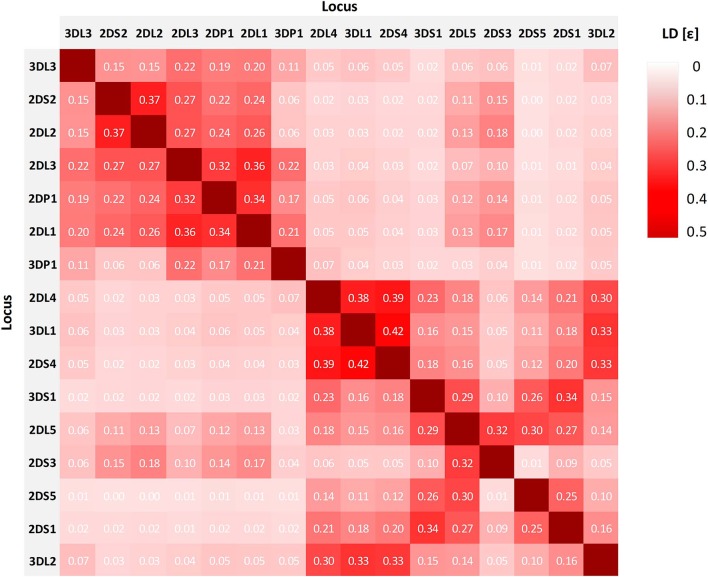
Overall linkage disequilibrium of 16 *KIR* loci in genomic orientation as designated by the normalized entropy difference ε. The value of ε ranges between 0 and 1, with larger values indicating stronger LD. Values of ε are displayed in the tiles.

Figure 4 shows the LD between the 16 analyzed loci of the *KIR* complex. The normalized entropy difference ε indicates a linkage disequilibrium within the designated centromeric and telomeric parts of the *KIR* gene complex, but much less between the two parts. For the pair of the two inner framework genes *KIR3DP1* and *KIR2DL4*, the value is close to equilibrium (ε = 0.07). The highest telomeric LD (ε = 0.42) is found for the loci *KIR3DL1* and *KIR2DS4*. In the vast majority of haplotypes, amongst others in the by far most frequent copy number haplotype P10_01(cA01~tA01), those two loci are adjacent and either both present or both absent. In addition, *KIR3DL1* is in LD with framework gene *KIR2DL4* (ε = 0.38), and *KIR2DL4* with *KIR2DS4* (ε = 0.39). In the centromeric stretch, the highest LD (ε = 0.37) is found for *KIR2DS2* and *KIR2DL2*, two adjacent and usually concurrent loci. A further block of linkage disequilibrium is formed by loci *KIR2DL3, KIR2DP1*, and *KIR2DL1* (0.32 ≤ ε ≤ 0.36). The three loci *KIR2DL5, KIR2DS3*, and *KIR2DS5* play a special role in the multi-locus LD analysis since our typing method does not allow the separation of *KIR2DL5A* and *KIR2DL5B* and thus the direct assignment of the *KIR2DL5*~*KIR2DS3*~*KIR2DS5* cluster to centromeric or telomeric position on the chromosome. The linkage analysis reveals LD for the two pairs *KIR2DL5*~*KIR2DS3* (ε = 0.32) and *KIR2DL5*~*KIR2DS5* (ε = 0.30), while the two loci *KIR2DS3* and *KIR2DS5* are almost in linkage equilibrium (ε = 0.01). Overall, the linkage of the three genes of this cluster is higher to loci of the telomeric than to loci of the centromeric region. *KIR2DS5* shows no linkage to genes of the centromeric *KIR* region at all. This is consistent with the conserved centromeric and telomeric allele combinations observed for the *KIR2DL5*~*KIR2DS3*~*KIR2DS5* cluster, according to which *KIR2DS5* is not present in centromeric position.

### Assignment of *KIR2DL5~KIR2DS3~KIR2DS5* to Centromeric or Telomeric Position on the Haplotype

The analysis of the allelic diversity of different copy number haplotype structures revealed the association of conserved allele combinations of gene cluster *KIR2DL5*~*KIR2DS3*~*KIR2DS5* with its localization on the chromosome (Supplementary Information S6). We applied this information to assign centromeric and telomeric positions of the gene cluster in our KIR HFs.

Of the 551 *KIR* allele group haplotypes, 164 contained the respective genes in a total of 11 different combinations. In 96.3% of the cases, the localization of the *KIR2DL5*~*KIR2DS3*~*KIR2DS5* genes could be assigned unambiguously. Six cases in two allele combinations included “NEW” alleles in *KIR2DS3*. For these cases we deduced the position of the clusters in the context of the overall haplotype structure. KIR HFs for the entire gene region with inferred localization of gene clusters *KIR2DL5*~*KIR2DS3*~*KIR2DS5* are shown in the Supplementary Information S8.

### Frequencies of Partial *KIR* Haplotypes

Estimation of *KIR* HFs of the centromeric gene region from *KIR* data of 838 parents resulted in 323 different allele group haplotypes with *f* ≥ 1/2*n* (frequency sum 97.1%), representing 22 partial copy number haplotypes (Supplementary Information S9). Frequency of the three structural haplotypes cA01, cB01, and cB02 reached a total of 92.8%. Assignment of cluster *KIR2DL5*~*KIR2DS3*~*KIR2DS* to the centromeric haplotypes was deduced as described above. Interestingly, the partial HFs of the centromeric *KIR* genes reveal an additional allele combination of the *KIR2DL5*~*KIR2DS3*~*KIR2DS5* cluster. One haplotype with the combination KIR2DL5^*^019B~ KIR2DS5^*^008 was found, the only centromeric occurrence of *KIR2DS5* in our study. Its frequency (*f* = 5.97^*^10^−4^) indicates a unique occurrence of the haplotype in our sample.

Accordingly, telomeric KIR HFs, including *KIR* data of 876 parents yielded 146 allele group haplotypes with *f* ≥ 1/2*n* (frequency sum 98.8%). Only 12 partial copy number haplotypes were represented. The frequency of copy number haplotypes tA01, tB01 (KIR2DS3 present), and tB01 (KIR2DS5 present) added up to 96.8% (Supplementary Information S10).

## Discussion

We present allele group *KIR* haplotypes estimated from 790 parents of a cohort of *n* = 403 German families. The restriction of haplotype structures to a set of copy number haplotypes from previous publications enabled us to cope with the enormous structural ambiguities of the *KIR* gene complex. Deducing diplotypes within the context of families allowed us to further reduce the number of possible diplotypes per individual. Our approach yielded a set of 551 allele group *KIR* haplotypes, representing 21 of the 92 CNPS haplotypes.

The results of our study are in good accordance with two other studies on allelic *KIR* haplotypes. A table comparing our Top20 haplotypes with findings of Vierra-Green et al. (44), Hou et al. (52) can be found in the Supplementary Information S11. Both former studies inferred their allelic HFs from cohorts of European ancestry without a family context and did not include information on the polymorphism of the pseudogene loci *KIR2DP1* and *KIR3DP1* in their haplotype determination. Haplotype structures were restricted to smaller reference sets of content haplotypes.

Nineteen of our Top 20 allele group *KIR* haplotypes correspond to allelic haplotypes published by Vierra-Green et al. (44). For the most frequent haplotype in both studies, KIR3DL3^*^003~KIR2DS2^*^001c~KIR2DL2^*^003~KIR3DP1^*^001c~KIR2DL4^*^001~KIR3DL1^*^002~KIR2DS4^*^001c~KIR3DL2^*^002c, we estimated a frequency of *f* = 0.0290, while Vierra-Green et al. determined *f* = 0.0306. During comparison of the allelic haplotypes it became apparent that several haplotypes in Vierra-Green et al. carried allele KIR2DS4^*^007 which never occurred in our haplotype set. Analysis of the sequence provided a potential explanation: KIR2DS4^*^007 differs from KIR2DS4^*^010 only in the first base of exon 4. As KIR2DS4^*^010 was not listed in the IPD-*KIR* database before version 2.2.0 (May 2010), this allele may not have been included in allele typing and interpretation of the former study. Our typing routine distinguishes between the two alleles and we find almost exclusively KIR2DS4^*^010 in our donors.

In a study published by Hou et al. (52), the centromeric and telomeric stretches of the *KIR* gene complex were analyzed separately and allelic haplotypes were grouped into different consensus structures. Our estimated allele group *KIR* haplotypes are in good agreement with their findings (Supplementary Information S7). The centromeric parts of 19 of our Top 20 allele group haplotypes were also described by Hou et al. They could be assigned to four of the five postulated centromeric consensus structures. In the case of the telomeric part, 18 of our Top 20 allele group haplotypes were described in the previous study. They account for 7 out of 8 suggested telomeric haplotype structures.

The comparison of our results to *KIR* haplotype data of non-European individuals shows clearly less similarities. A recent study on *HLA* and *KIR* diversity in individuals from African populations reveals differences in the *KIR* HFs between the seven populations from Western, Central, and Eastern Africa (66). The African haplotypes furthermore show a different allelic distribution than those of our German cohort. Some of the alleles show exclusive occurrence in one of the geographically distinct cohorts. Alleles 3DL3^*^001 or 3DL1^*^002, which are, for example, frequent in our sample, were not observed in the African samples. Vice versa, the African cohorts contain frequent alleles that are not found in our German haplotypes, e.g., 3DL3^*^005 or 3DL1^*^017. The centromeric part of only seven and the telomeric part of 10 of our Top 20 allele group haplotypes are present in the African cohorts.

Two levels of linkage are visible in our LD analyses. On the one hand, observed LD in pairs of KIR loci clearly reflect the structural constraints of *KIR* haplotypes by echoing proximity of loci as given in the different CNPS haplotypes. On the other hand, the linkage of certain allele groups within these structural constraints indicates a non-stochastic distribution of alleles to particular haplotypes. The low deviation from linkage equilibrium between genes of the centromeric and the telomeric gene stretch supports the assumption of a recombination hot spot between *KIR3DP1* and *KIR2DL4*. Our findings on LD confirm data from previous research (44). Our set of 32 allele group LD pairs includes 20 of the 26 two-allele haplotypes that showed significant LD in the former study.

The genetic linkage of alleles within given haplotype structures reflected in the LD analysis is confirmed when allele group haplotypes are clustered by their underlying copy number haplotype. The analysis reveals two interesting results: First, allelic variability in our cohort is found to be higher in A than in B haplotypes. This observation agrees with previous descriptions (48) and is also in accord with findings of lower overall allelic diversity in the activating compared to the inhibitory loci (44, 53). And second, we observe conserved allelic correlations specific to five distinct centromeric and telomeric haplotype motifs (cA01, cB01, cB02, tA01, and tB01). The detected differences in allelic variability and the correlation of certain alleles to A and B haplotype motifs also comprises the framework genes. These results indicate a *KIR* gene inheritance in closely linked blocks with a recombination hot spot between the genes *KIR3DP1* and *KIR2DL4*. The very particular allelic linkage within the *KIR2DL5*~*KIR2DS3/2DS5* haplotype enabled us to assign this gene cluster to centromeric or telomeric position in our KIR haplotypes even though our typing method did not allow a differentiation between the two loci KIR2DL5A and KIR2DL5B. Beyond that, the observed allele group haplotype patterns of the telomeric and centromeric *KIR2DL5*~*KIR2DS3/2DS5* clusters correspond to those described in previous publications on individuals of European ancestry (52, 60).

The knowledge of family relations in our cohort substantially reduced the number of possible diplotypes per individual. This had two consequences. First, it increased the number of individuals who were excluded from the *KIR* HF estimation due to the absence of a valid combination of CNPS haplotypes that explained their diplotypes. This demonstrates, on the one hand, the incompleteness of our used CNPS haplotype list. On the other hand, it reveals the limitation of *KIR* HF estimation without family context, where, in consequence, the same *KIR* genotypes were described with at least one incorrectly assigned pair of CNPS haplotypes. And second, the reduced number of possible diplotypes per individual provided a reasonable basis for a successful execution of an EM algorithm. Apart from reducing phasing ambiguities, the family context further offers the potential to detect typing inaccuracies and recombination events that would remain hidden otherwise.

Given the multiple constraints we applied to our *KIR* data in order to estimate allele group *KIR* HFs, several sources of possible bias to our results have to be considered. One important bias may be caused by the restriction of *KIR* haplotypes to a limited copy number pattern set. For 10.6% of the parents in our cohort, the deduced diplotypes could not be explained by a pair of haplotypes from the CNPS, indicating further *KIR* complexity on the copy number level and the need for extension of the CNPS. The estimation of partial (centromeric and telomeric) haplotypes of the same cohort led to a reduction of this percentage of excluded individuals. This indicates that the missing structural diversification of the pattern haplotypes is mostly limited to variation in sub-ranges of the KIR gene complex. Thorough analysis of the diplotypes of the excluded individuals by alternative typing methods beyond the routine high-throughput *KIR* genotyping in the context of unrelated HSCT donor registration (53) will be of great importance for the future refinement of our approach. Such extended analyses could also detect hybrid genes that are not described in the IPD-*KIR* database to which our routine typing method is blind, but go beyond the scope of the present study. Further bias may be introduced by sequencing or sequence interpretation errors. However, the impact of this potential bias should be minimal with our approach because the low overall error rate of the NGS high throughput platform was further reduced by verification of the *KIR* data within the families. The accuracy of the high throughput *KIR* analysis in a curated sample was found to exceed 99% (53). In the 13 remaining cases of mismatching loci and one presumable case of recombination in our cohort, where typing errors could not be excluded as a cause without re-typing of the respective family members, parents were excluded from the HF estimation. Moreover, the restriction of the cohort to individuals with German origin needs to be treated with some caution. This information was collected via self-assessment of the volunteer stem cell donors during the registration process. The perception of the term “origin” is quite individual (67) and in many cases it is simply difficult to describe an individual's origin by means of one single country code. Finally, family lineage was not known for the individuals of our cohort but deduced from concordance of surname and address and a defined age difference between presumed parents and offspring. However, the verification of genetic relationship via the *HLA* genes and exclusion of families with any mismatching loci in the *KIR* genes from the final HF estimation should compensate for lack of direct family information.

In conclusion, our approach yielded a set of 551 allele group *KIR* haplotype frequencies from a German cohort that is in good accordance with results from other groups. We provide additional data on the diversification of *KIR* haplotypes by inclusion of allelic polymorphisms of pseudogenes *KIR2DP1* and *KIR3DP1*. The use of family information during diplotype deduction allowed the exclusion of incorrect phasing variants. Our *KIR* haplotype frequencies reveal relations between *KIR* copy number haplotypes and allele frequencies, which will be a valuable basis for future research. The application of this approach, e.g., to larger cohorts of different ethnic origin, will further broaden our knowledge and understanding of the very complex nature of the *KIR* genes.

## Research Data

DNA was extracted from blood samples or buccal swabs with the informed consent of the donors. Research data used in this publication was collected from the data subjects and processed on the basis of an informed consent in accordance with the EU Data Protection Regulation (EU-GDPR). Data subjects agreed to their data being processed for scientific studies, in particular with the aim to improving the treatment of patients with blood cancer and other life threatening diseases. The publication itself does not include identifiable personal data.

## Data Availability Statement

The raw data supporting the conclusions of this article will be made available by the authors, without undue reservation, to any qualified researcher.

## Author Contributions

JSa and US conceived the project. US designed and implemented algorithms, carried out analyses, and wrote the first draft of the manuscript. GS, CM, AH, MK, DS, and JP analyzed and interpreted KIR genotyping data. All authors contributed to manuscript revision, read, and approved the submitted version.

### Conflict of Interest

The authors declare that the research was conducted in the absence of any commercial or financial relationships that could be construed as a potential conflict of interest.

## Abbreviations

CNPS: copy number haplotype pattern set
EM: expectation-maximization
GvHD: graft-versus-host disease
HF: haplotype frequency
HLA: human leukocyte antigen
HSCT: hematopoietic stem cell transplantation
KIR: Killer-cell immunoglobulin-like receptor
LD: linkage disequilibrium
MHC: major histocompatibility complex
NK: natural killer.
